# The Importance of Perinatal Follow-Up in the Management of Critical Congenital Heart Diseases: A Pediatric Heart Center Experience

**DOI:** 10.3390/children12060767

**Published:** 2025-06-13

**Authors:** Halise Zeynep Genc, Demet Oguz, Mehmet Gumustas, Dilek Yavuzcan Ozturk, Kubra Kurt Bilirer, Ibrahim Polat, Merih Cetinkaya, Ali Can Hatemi, Erkut Ozturk

**Affiliations:** 1 Department of Pediatric Cardiology, Saglik Bilimleri University Basaksehir Cam and Sakura Hospital, Istanbul 34480, Turkey; mehmet.gumustas1@saglik.gov.tr (M.G.); erkut.ozturk@sbu.edu.tr (E.O.); 2 Department of Neonatology, Saglik Bilimleri University Basaksehir Cam and Sakura Hospital, Istanbul 34480, Turkey; demet.oguz1@saglik.gov.tr (D.O.); d.yavuzcanozturk@saglik.gov.tr (D.Y.O.); merih.cetinkaya@saglik.gov.tr (M.C.); 3 Department of Perinatology, Saglik Bilimleri University Basaksehir Cam and Sakura Hospital, Istanbul 34480, Turkey; kubra.kurtbilirer@saglik.gov.tr (K.K.B.); ibrahim.polat3@saglik.gov.tr (I.P.); 4 Department of Pediatric Cardiovascular Surgery, Saglik Bilimleri University Basaksehir Cam and Sakura Hospital, Istanbul 34480, Turkey; alican.hatemi@sbu.edu.tr

**Keywords:** newborn, critical congenital heart disease, perinatal

## Abstract

**Objectives**: In the neonatal period, 25% of cases with critical congenital heart disease (CCHD) require surgical or interventional palliative and corrective procedures. Prenatal diagnosis and timely intervention can positively impact neonatal mortality and morbidity. This study evaluated the effects of perinatal follow-up on the management of CCHD. **Methods**: The study was conducted on term neonates diagnosed with CCHD, who were monitored in the neonatology and pediatric cardiac intensive care unit between 1 January 2023 and 1 January 2024. The cases were categorized into CCHD with prenatal follow-up (Group I), CCHD born without follow-up at our hospital (Group II), and CCHD accepted from external centers (Group III). Neonatal mortality and morbidity outcomes of these cases that underwent surgical or interventional procedures were statistically evaluated. **Results**: During the study period, there were 280 neonatal cases (50% male). Among these cases, 30% were in Group I (*n* = 84), 20% in Group II (*n* = 56), and 50% in Group III (*n* = 140). The cesarean section rate was higher in Group I compared to the other groups (80% vs. 52% vs. 45%), and the preoperative lactate levels were lower (0.9 vs. 1.7 vs. 2.1). The anatomical diagnoses, ventricular physiology, operation time, and interventional procedure time were similar. After interventional or surgical procedures, morbidity (22% vs. 25% vs. 36%) and mortality rates (6% vs. 9% vs. 18%) were lower in Group I and Group II compared to Group III. **Conclusions**: All infants diagnosed with CCHD before birth should be delivered in a tertiary heart center, which positively contributes to neonatal mortality and morbidity. More effort is needed to improve prenatal screening programs.

## 1. Introduction

The frequency of congenital heart disease (CHD) ranges from 8 to 10 per thousand live births and constitutes a significant health issue for term infants. Newborns with CHD may have some nonspecific symptoms such as fatigue while feeding, failure to gain weight, cyanosis, and rapid breathing. One third of CHD are in the critical group and require catheter intervention or cardiac surgery within the first 28 days of life. Critical congenital heart diseases (CCHD) typically appear with these nonspecific symptoms immediately after birth or within the first week of life [1]. Low APGAR scores are also associated with neonates with CHD, and these patients are more likely to experience a complicated fetal-to-neonatal transition [2].

The distribution of CCHD shows some differences from that of overall CHD cases. CHD can be divided into the four groups, according to treatment approach and anatomical features, as follows: diseases dependent on systemic ductal flow (hypoplastic left heart syndrome, aortic stenosis, and interrupted aortic arch), diseases dependent on pulmonary ductal flow (tetralogy of Fallot, pulmonary atresia, critical pulmonary stenosis, and Ebstein anomaly), diseases requiring adequate mixing (transposition of great arteries), and others (total anomalous pulmonary venous drainage and truncus arteriosus) [3,4,5].

Prenatal diagnosis gives parents time to consider the pros and cons of continuing the pregnancy versus terminating it. It also helps improve survival outcomes in CHDs by ensuring timely and appropriate treatment. For those who choose to continue with the pregnancy, ongoing monitoring and observation of disease progression may be performed [6].

Several studies from high-income countries have shown the beneficial impact of the prenatal diagnosis of CCHDs in improving perioperative outcomes. However, in developing countries, there is limited information on this topic, and the results regarding the impact of prenatal diagnosis are inconsistent [6,7,8].

In this study, we compared the early mortality and morbidity rates of three different groups of critically ill newborns with CHD: those diagnosed prenatally at our clinic, those diagnosed postnatally at our clinic, and those transferred from other centers. Additionally, risk factors affecting these rates were investigated.

## 2. Materials and Methods

The study was conducted prospectively on term newborns diagnosed with CCHD who were monitored in the neonatal and pediatric cardiac intensive care unit (ICU) between 1 January 2023 and 1 January 2024. The study was conducted in accordance with the Declaration of Helsinki after the approval from the local ethics committee.

Premature infants (<37 weeks), newborns with stable CHD that did not require neonatal intervention, and conditions requiring only medical treatment (such as arrhythmias, myocarditis, etc.) were excluded from the study.

We have standardized our practices in our current approach for the perinatal management of cases. In our center, CCHD is usually diagnosed at approximately 21–22 weeks of gestation, during the detailed second-trimester fetal anatomical scan. Particularly in cases of complex cardiac anomalies with single ventricle morphology, the diagnosis can be made during the late first trimester (12–14 weeks) or early second trimester (16–18 weeks) through early fetal echocardiography. The timing of diagnosis varies depending on the type of cardiac anomaly; anomalies such as hypoplastic left heart syndrome and atrioventricular septal defects are usually identified earlier, while progressive lesions like coarctation of the aorta may be diagnosed later.

The most preferred imaging modality for prenatal diagnosis is high-resolution fetal echocardiography, which provides detailed anatomical evaluation. In cases with suspected extracardiac anomalies or complex findings, fetal MRI may be used as an adjunct imaging tool to support prenatal counseling and postnatal planning.

Once a prenatal diagnosis of CCHD is made, a multidisciplinary perinatal follow-up protocol is initiated. The frequency of follow-up varies depending on the complexity of the cardiac anomaly and the presence of associated anomalies; however, perinatology visits are typically scheduled every 2 to 3 weeks. After the diagnosis, fetal echocardiography is repeated at least twice and more frequently if progression of the anomaly is suspected or if additional obstetric complications are present.

Birth planning is individualized for each patient but is mostly determined by the presence of obstetric comorbidities. Pregnant women without obstetric comorbidity who are followed up because of a fetus with CHD are followed up until 40–41 weeks of gestation like normal pregnant women. In cases of poor prognosis, such as complex cardiac anomalies with single ventricle morphology, severe extracardiac malformations, confirmed genetic syndromes, or chromosomal disorders, pregnancy termination is discussed in multidisciplinary counseling. In accordance with national legal regulations, the option of termination is offered to the family.

Due to the structure of our hospital, there are night shift staff from pediatric cardiology, neonatology, and perinatology clinics every day. When a pregnant woman is taken to the delivery room, the perinatology department informs the neonatology and pediatric cardiology departments. A message is sent to the clinics regarding the birth of a newborn with CHD through the hospital automation system. Echocardiography is provided in the delivery room, and the newborn is quickly evaluated by the pediatric cardiologist after birth within the first hour of life. If necessary, prostaglandin infusion is started immediately. During antenatal follow-up, patients who may need early intervention are identified, and the catheterization laboratory or operating room is prepared accordingly. In our center, the delivery room, neonatology, and pediatric cardiology department, which includes the catheterization laboratory and operating room, are all connected and close to each other, and interventions can be performed quickly when needed.

**Figure 1:**
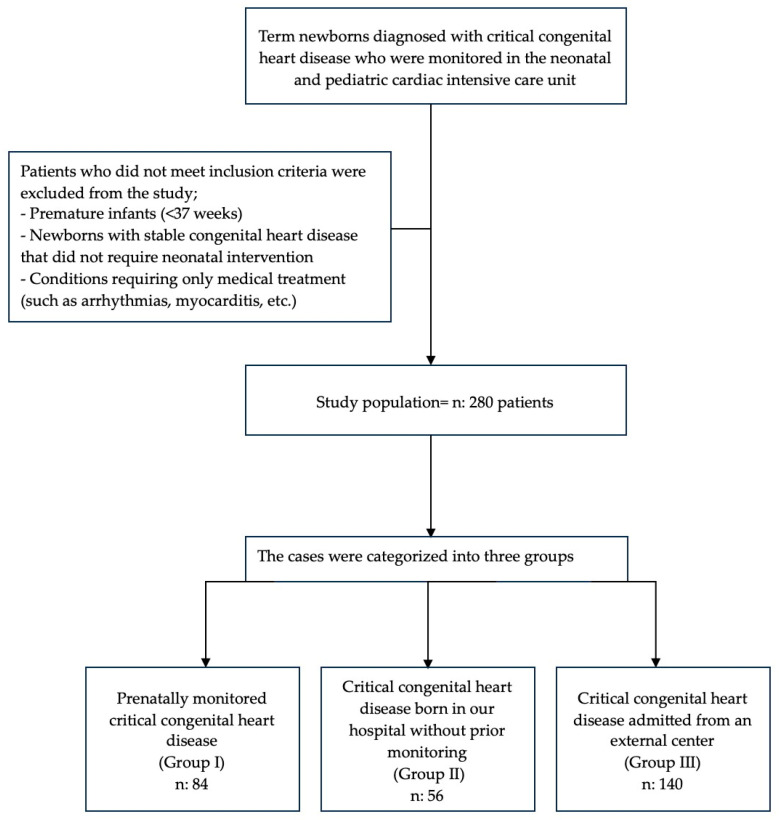
Flowchart of patient enrollment and exclusion criteria.

A total of 280 cases were categorized as follows: prenatally monitored critical congenital heart disease (Group I), critical congenital heart disease born in our hospital without prior monitoring (Group II), and critical congenital heart disease admitted from an external center (Group III) (Figure 1).

For Group I, which includes patients with CCHD who are followed up in the perinatology clinic of our hospital, a database was created to include all demographic and clinical data, cardiac diagnosis, non-cardiac anomalies, consultations, and details of postnatal care. A similar approach was used to record demographic and clinical data, as well as cardiac diagnosis, for Group II, which includes patients with CCHD born in our hospital without antenatal follow-up. Group III patients were initially admitted to other hospitals, diagnosed there, and then transferred to our center with suspected CCHD. The clinical condition at the referring hospital’s admission and the details of stabilization measures taken were not available for all cases. For these cases, we recorded the mode of transfer (monitored/unmonitored) and the transfer distance.

The recorded demographic details included gestational age at birth, type and place of delivery, birth weight, age and weight at admission, and gender. The definitive cardiac diagnosis was determined and recorded using echocardiography. For newborns who underwent surgical repair, the cardiac diagnosis was further categorized according to both the STAT and RACHS-1 risk classification systems. The STAT (Society of Thoracic Surgeons–European Society for Cardiothoracic Surgery) score is a risk scoring system designed to analyze the risk of mortality from congenital heart surgery procedures. It contains a total of 5 categories, organized so that procedures associated with the lowest mortality rates are in Category 1 and procedures associated with the highest mortality rates are in Category 5. The risk adjustment for congenital heart surgery (RACHS-1) score was created to determine the risk of death after CHD surgery for pediatric patients under 18 years of age. RACHS-1 groups procedures into 6 levels of increasing risk of mortality [7,8,9]. The Preoperative Cardiac and Hemodynamic Status Assessment (PRACHS) score evaluates 15 different clinical and laboratory parameters. Each parameter was considered normal if it was within standard reference ranges and was given a score of 0; if abnormal, it was given a score of 1 [5]. The preoperative or pre-angiography condition of the three study groups was assessed using the Pre-operative Assessment of Cardiac and Hemodynamic Status (PRACHS) score.

This evaluation was conducted for all three study groups following the newborn’s admission to our pediatric cardiac ICU.

The following outcome variables were included and compared between the three groups. Preoperative clinical status was evaluated using the scoring systems described above. Postoperative outcomes were evaluated by morbidity, mortality, ventilation duration, and intensive care unit and hospital stay.

Data were analyzed using SPSS Statistics for Windows (version 23: SPSS Inc., Chicago, IL, USA). The median with the interquartile range (IQR) was used to describe continuous data, whereas the absolute count with percentages was used for categorical data. The Pearson chi-squared test and one-way analysis of variance (ANOVA) were used to compare variables between the groups. Statistical significance was set at *p* < 0.05.

## 3. Results

During the study period, there were 280 neonatal cases (50% male). Among these cases, 30% were in Group I (*n* = 84), 20% in Group II (*n* = 56), and 50% in Group III (*n* = 140).

The main cardiac diagnoses of the cases are shown in Table 1.

The postnatal evaluation confirmed the prenatal diagnosis accurately in 67 cases (79%), with minor variations resulting in no change to treatment in 24 cases (16.6%) and major variations in three cases (3.5%). The diagnoses of the three cases with major variation were as follows; in one case, the antenatal diagnosis was hypoplastic left heart syndrome, while the postnatal diagnosis was total anomalous pulmonary venous drainage (TAPVD). In the second case, the antenatal diagnosis was double inlet left ventricle–pulmonary atresia, while the postnatal diagnosis was double inlet right ventricle–pulmonary atresia. In the third case, the antenatal diagnosis was right isomerism, complete atrioventricular septal defect, and double outlet right ventricle, while the postnatal diagnosis revealed TAPVD in addition to these diagnoses. These cases showed us that the prenatal diagnosis of TAPVD is particularly difficult.

The demographics and patient characteristics are shown in Table 2.

**Figure 2:**
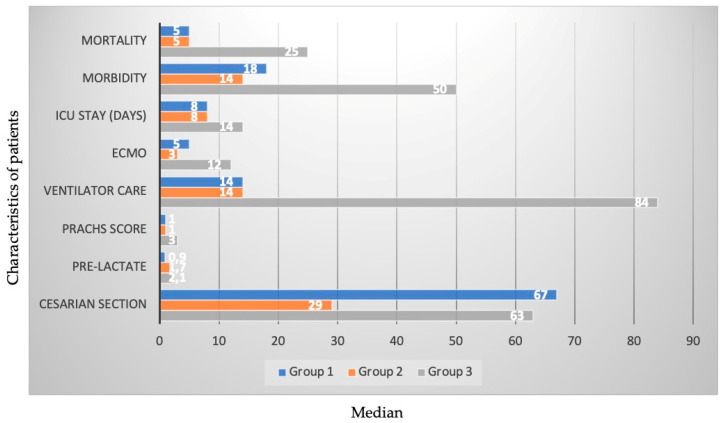
The characteristics with the most statistically significant difference.

The cesarean section rate was higher in Group I compared to the other groups (80% vs. 52% vs. 45%), and preoperative lactate levels were lower (0.9 vs. 1.7 vs. 2.1). The median PRACHS score was significantly higher in Group III compared to the other groups (1 vs. 1 vs. 3). The ventilator care was higher in Group III compared to the other groups (14 vs. 14 vs. 84). Days staying in the intensive care unit were higher in Group III compared to the other groups (8 vs. 8 vs. 14). The extracorporeal membrane oxygenation (ECMO) duration was higher in Group III compared to the other groups (5 vs. 3 vs. 12). The ventricular physiology and interventional procedure time of the cases were similar. There was no significant difference between arrhythmias, acute kidney injury, low cardiac output syndrome (LCOS), and days of postop hospital stay. After interventional or surgical procedures, morbidity (22% vs. 25% vs. 36%) and mortality rates (6% vs. 9% vs. 18%) were lower in Group I and Group II compared to Group III. The characteristics with the most statistically significant difference are given in Figure 2.

## 4. Discussion

In this study, early outcomes of newborns diagnosed with CCHD who were diagnosed prenatally, diagnosed postnatally, or transferred from an external center were evaluated at a tertiary cardiac center. It was found that newborns with a prenatal diagnosis of CCHD (Group I) had lower morbidity and mortality rates compared to other patients. This is a valuable study as it is a large-scale prospective study that emphasizes the importance of improving prenatal diagnoses in a developing country.

CCHD continue to be a major cause of morbidity and mortality in infants. Most cases can be detected in the second trimester of pregnancy with level 2 obstetric ultrasound, and CHD can be confirmed with a detailed fetal echocardiogram. Cardiac screening during the anatomy scan in low-risk pregnancies has variable detection rates. However, when a referral for a fetal echocardiogram is made, the diagnostic rate is excellent. In recent years, the rate of detection of CCHD in the fetal period has increased. In their series, Cloete and colleagues [10] reported a 47% detection rate of CCHD between 2011 and 2014, while Chakraborty and colleagues [11] identified 68.1% of patients with CCHD through prenatal ultrasound over a 10-year period. In our study, a prenatal diagnosis was made in 60% (84/140) of the infants born in our hospital. The fact that a significant portion of undiagnosed cases had few perinatology clinic visits may have contributed to this.

Many studies have emphasized the positive impact of prenatal diagnosis on the preoperative status of patients. The impact of the socioeconomic conditions of countries on these outcomes is undeniable. In developed countries, the standard application of fetal ultrasonography, early physical examination, and the use of pulse oximetry for newborn cardiac screening, as well as the rapid transfer of cases with CCHD, have positively affected the outcomes. On the contrary, in developing countries, the insufficient development of prenatal diagnosis and perinatal care may negatively affect the outcomes [12]. We also found that the outcomes were worse in patients transferred to us from an external center (Group 3). Since we are among the developing countries, the stabilization and transfer conditions of patients with a diagnosis of CHD may not always be optimal. In addition, since antenatal fetal cardiac evaluation is not possible in every center of a developing country, newborns who may need intervention in the early postnatal period (such as TGA with a restrictive interatrial septum) cannot be detected and consequently cannot be intervened, resulting in high morbidity and mortality rates.

Thakur and colleagues [13] evaluated 129 cases diagnosed with critical duct-dependent cardiac lesions (63 cases with prenatal diagnosis and 66 cases with postnatal diagnosis). Unlike cases with postnatal diagnosis, they observed no deaths (0/63 vs. 5/66; *p* = 0.06) or cardiac arrests (0/63 vs. 9/63; *p* = 0.003) before surgery or catheter intervention in cases with a prenatal diagnosis of CCHD. They found that neonates with a fetal diagnosis were admitted to the hospital earlier (median 0 (range 0–3) vs. 2 (0–25) days; *p* < 0.001) and were less likely to require preoperative ventilation (19/63 vs. 31/61; *p* = 0.03) and vasoactive medication (4/63 vs. 15/61, *p* = 0.006) compared to neonates with postnatal diagnosis. Quartermain and colleagues [14], in their study of 12,899 neonates with CCHD from 112 centers, reported that there are lower rates of major preoperative risk factors in the prenatal diagnosis group. In the study by Vijayaraghavan and colleagues [5], the preoperative status was significantly better in the prenatal group. In our study, the preoperative clinical conditions of the cases referred from outside the hospital were found to be worse, in line with the above-mentioned studies.

Different outcomes have been reported in the literature regarding the effects of prenatal diagnosis and planned perinatal care on perioperative outcomes. In the study by Guvenc and colleagues [15], it was found that cases with transposition of the great arteries who received prenatal diagnoses had lower mortality and morbidity rates. Kumar et al. demonstrated that the prenatal diagnosis of hypoplastic left heart syndrome and transposition of the great arteries is not associated with improvements in early postoperative outcomes [16]. Sivarajan et al. observed that prenatal diagnosis resulted in an improvement in preoperative clinical status but had no effect on mortality [17].

In our study, cases with prenatal diagnosis had significantly lower mortality and morbidity rates compared to other groups. At this point, it can be considered that the particularly poor clinical condition of the transferred babies was influential.

### Limitations

The main limitations of the study include the possibility of referral bias and the impact of institutional expertise on postoperative outcomes. The preoperative condition of infants diagnosed after birth (Group II and Group III) was assessed only after their admission to our intensive care unit. It is highly likely that some infants in Group III were stabilized at the referring hospital and arrived in a better condition than at their initial presentation. The absence of differences in postoperative mortality and morbidity measurements may reflect institutional bias and expertise.

## 5. Conclusions

Delivering all newborns diagnosed with critical congenital heart disease in a tertiary cardiac center contributes positively to neonatal mortality and morbidity outcomes. More efforts are needed to improve prenatal screening programs.

## Figures and Tables

**Table 1:** Primary cardiac diagnoses.

Diagnosis	Group I: *n* = 84	Group II: *n* = 56	Group III: *n* = 140
TGA	23	9	29
TOF	7	5	13
HLHS	18	11	25
TAPVD	4	7	8
DORV	3	2	7
VSD + PA	9	5	19
CoA or IAA or Hypoplastic aortic arch	11	4	20
DILV	2	3	5
Other	7	8	14

CoA: coarctation; DILV: double inlet left ventricle; DORV: double outlet right ventricle; HLHS: hypoplastic left heart syndrome; IAA: interrupted aortic arch; PA: pulmonary atresia; TAPVD: total anomalous pulmonary venous drainage; TGA: transposition of the great arteries; TOF: tetralogy of Fallot; VSD: ventricular septal defect.

**Table 2:** Demographics and patient characteristics.

Variables	Group I: *n* = 84	Group II: *n* = 56	Group III: *n* = 140	*p*
Weight, kg	3 (2.8–3.2)	3.1 (2.9–3.4)	2.9 (2.7–3.2)	NS
Male	41 (49)	29 (51)	70 (50)	NS
Cesarian section	67 (80)	29 (52)	63 (45)	**0.02**
Single ventricle physiology	29 (34.5)	24 (42.8)	48 (34.2)	NS
Cyanotic heart disease	47 (56)	28 (50)	72 (51.4)	NS
Pre-lactate mmol/L	0.9 (0.6–1.3)	1.7 (1.5–2)	2.1 (1.9–2.4)	**<0.001**
PRACHS score	1 (0–2)	1 (0–2)	3 (2–4)	**<0.001**
Ventilator care	14 (16.6)	14 (25)	84 (60)	**<0.001**
Intervention or operation	71 (84)	45 (81)	112 (80)	NS
RACHS-1 ≥ 4	47 (50)	17 (30)	56 (40)	NS
STAT	3 (2–4)	3 (2–4)	3 (2–4)	NS
ECMO	5 (5.9)	3 (5.5)	12 (8.5)	**0.04**
Arrhythmias	7 (8.3)	5 (8.9)	14 (10)	NS
Acute kidney injury	13 (15.4)	9 (16)	28 (20)	NS
LCOS	17 (19.4)	11 (19.6)	35 (25)	NS
ICU stay (days)	8 (6–10)	8 (6–10)	14 (10–16)	**0.008**
Postop hospital stay (days)	16 (12–20)	18 (15–24)	24 (20–28)	NS
Morbidity	18 (22)	14 (25)	50 (36)	**<0.001**
Mortality	5 (6)	5 (9)	25 (18)	**<0.001**

Median (IQR). ECMO: extracorporeal membrane oxygenation; ICU: intensive care unit; LCOS: low cardiac output syndrome; NS: non-significant; PRACHS: Pre-operative Assessment Of Cardiac And Hemodynamic Status; RACHS-1: risk adjustment for congenital heart surgery; STAT: The Society of Thoracic Surgeons–European Association for Cardio-Thoracic Surgery.

## Data Availability

The datasets used and/or analyzed during the current study are available from the corresponding author upon reasonable request due to privacy.

## Abbreviations

The following abbreviations are used in this manuscript:

ANOVA	Analysis of variance
COA	Coarctation
CCHD	Critical congenital heart disease
CHD	Congenital heart disease
CPB	Cardiopulmonary bypass
DILV	Double inlet left ventricle
DORV	Double outlet right ventricle
ECMO	Extracorporeal membrane oxygenation
HLHS	Hypoplastic left heart syndrome
IAA	Interrupted aortic arch
ICU	Intensive care unit
LCOS	Low cardiac output syndrome
NS	Non-significant
PA	Pulmonary atresia
PRACHS	Pre-operative assessment of cardiac and hemodynamic status
RACHS-1	Risk adjustment for congenital heart surgery
STAT	The Society of Thoracic Surgeons–European Association for Cardio-Thoracic Surgery
TAPVD	Total anomalous pulmonary venous drainage
VSD	Ventricular septal defect
